# P01.12. Prophylactic effects of Lonicera japonica extract on dextran sulfate sodium-induced colitis in a mouse model by inhibition of the Th1/Th17 response

**DOI:** 10.1186/1472-6882-12-S1-P12

**Published:** 2012-06-12

**Authors:** J Park, J Kim, B Ryu, S Ko, S Jongki, Y Bu

**Affiliations:** 1 College of Oriental Medicine, Kyung Hee University, Seoul, Republic of Korea

## Purpose

Inflammatory bowel diseases (IBDs) are chronically relapsing inflammatory disorders of the intestine. Although some therapeutic agents, including steroids, are available for treatment of IBD, these agents have limited use. Therefore, dietary supplements have emerged as possible interventions for IBD. Japanese honeysuckle flower, the flower of Lonicera japonica, is a well-known dietary supplement and has been used to prevent or treat various inflammatory diseases.

## Methods

In the current study, we investigated the effects of L. japonica on experimental murine colitis. Colitis was induced by 5% dextran sulfate sodium (DSS) in Balb/c mice. The water extract of L. japonica (LJE) at doses of 20, 100 or 500 mg/kg was orally administered to the mice twice a day for 7 days. Body weight, colon length and a histological damage score were assessed to determine the effects on colitis. Cytokine profiles were assessed to examine the effects on T helper (Th) cell-related immunological responses. In addition, CD4 + CD25 + FOXP3 T cells were analyzed *in vivo* and *in vitro* for investigating the effects on regulatory T (T_reg_) cells.

## Results

LJE showed dose-dependent inhibitory effects against colon shortening, weight loss and histological damage. LJE down-regulated interleukin (IL)-1β, tumor necrosis factor α, interferon γ, IL-6, IL-12 and IL-17. However, LJE did not show any significant effects on IL-10, IL-23, transforming growth factor β1 and T_reg_ cell populations.

## Conclusion

In conclusion, LJE showed protective effects against DSS-induced colitis via the Th1/Th17 pathway and not via T_reg_ cell-related mechanisms.

